# The effect of very low dose pulsed magnetic waves on cochlea^☆^

**DOI:** 10.1016/j.bjorl.2018.10.013

**Published:** 2018-12-12

**Authors:** Birgül Tuhanioğlu, Sanem Okşan Erkan, Seren Gülşen Gürgen, Talih Özdaş, Orhan Görgülü, Figen Çiçek, İsmail Günay

**Affiliations:** a Health Science University Adana City Hospital, Department of Otorhinolaryngology-Head and Neck Surgery, Adana, Turkey; b Celal Bayar University, Department of Histology and Embriology, Manisa, Turkey; c Çukurova University, Department of Biophysics, Adana, Turkey

**Keywords:** Electromagnetic field, Otoacoustic emissions, Cochlea, Campo eletromagnético, Emissões otoacústicas, Cóclea

## Abstract

**Introduction:**

In daily life biological systems are usually exposed to magnetic field forces at different intensities and frequencies, either directly or indirectly. Despite negative results, the therapeutic use of the low dose magnetic field has been found in recent studies. The effect of magnetic field forces on cochlear cells is not clear in the literature.

**Objective:**

In our study, we first applied in vivo pulsed magnetic fields to laboratory rats to investigate the effects on cochlea with distortion product otoacoustic emission test followed by histopathological examinations.

**Methods:**

Twelve rats were included in this study, separated into two groups as study group and control group. The rats in the study group were exposed to 40 Hz pulsed magnetic field for 1 h/day for 30 days; the hearing of the rats was controlled by otoacoustic emission test. Also, their cochleas were removed and histochemical examination was performed by Caspase-3, Caspase-9, and TUNEL methods.

**Results:**

A statistically significant difference was determined (*p* < 0.05) when the hearing thresholds of the groups obtained by using 5714 Hz and 8000 Hz stimuli were compared by Kruskal–Wallis test. A significant reaction was observed in the study group, especially in the outer ciliated cells during immunohistochemical examinations by using Caspase-3 and Caspase-9 methods. A significantly positive difference was determined in the study group, especially at the outer ciliated cells and the support cells of the corti organ, when compared to the control group (*p* < 0.05) by the TUNEL method.

**Conclusion:**

According to the results of our study, the very low dose magnetic field, which is considered to be used for therapeutic purposes recently, can cause both auditory function defects and histopathologic damage in cochlear cells.

## Resumo

**Introdução:**

Os sistemas biológicos são geralmente expostos a forças de campo magnético em diferentes intensidades e frequências, direta ou indiretamente, na vida diária. Apesar dos resultados negativos, o uso terapêutico do campo magnético de baixa dose tem sido encontrado em estudos recentes. O efeito das forças do campo magnético sobre as células cocleares não está claro na literatura.

**Objetivo:**

Em nosso estudo, aplicamos pela primeira vez campos magnéticos pulsados *in vivo* em ratos de laboratório para investigar os efeitos na cóclea através do teste de emissão otoacústica por produto de distorção e análises histopatológicas.

**Método:**

Doze ratos foram incluídos neste estudo, os quais foram separados em dois grupos, grupo de estudo e grupo controle. Os ratos do grupo de estudo foram expostos a campo magnético pulsado de 40 Hz por 1 hora/dia por 30 dias, e a audição dos ratos foi controlada por testes de emissão otoacústica. Além disso, suas cócleas foram colhidas e o exame histoquímico foi feito pelos métodos caspase-3, caspase-9 e TUNEL.

**Resultados:**

Foi determinada uma diferença estatisticamente significante (*p* < 0,05) quando os limiares auditivos dos grupos obtidos por meio dos estímulos de 5714 Hz e 8000 Hz foram comparados pelo teste de Kruskal-Wallis. Uma reação significante foi observada no grupo de estudo, especialmente nas células ciliadas externas nas análises imuno-histoquímicas, com os métodos caspase-3 e caspase-9. Uma diferença significantemente positiva foi determinada no grupo de estudo, especialmente nas células ciliadas externas e nas células de suporte do órgão de Corti, quando comparadas com o grupo controle (*p* < 0,05) pelo método TUNEL.

**Conclusão:**

De acordo com os resultados do nosso estudo, o campo magnético de dose baixa, que tem sido considerado para uso terapêutico recentemente, pode causar defeitos na função auditiva e danos histopatológicos nas células cocleares.

## Introduction

Electromagnetic (EM) waves are the spreading of energy with the characters of both wave and particles. Thus, the spectrum formed by considering the wave characteristics and the energies of EM waves is called the electromagnetic spectrum.^1^

EM waves are classified into two groups, ionized and non-ionized ones. Non-ionized ones are classified as UV, visible light, infrared radiation, microwave, and radio frequency according to decreasing energy. Radio waves are at the bottom of this spectrum.^1^ Since the radio waves cover a very wide area of the spectrum, they are separated into 11 sub-units with respect to the increase in certain frequency values. Extremely Low Frequency (ELF) EM fields which we have used in our study are generated by the power sources operating on mains frequency. Exteremely Low and Low frequency pulsed magnetic field is recently and frequently been used in many treatments like neuropathy therapy, bone marrow edema, promoting bone formation after fractures and wound healing in different departments.^2^, ^3^, ^4^, ^5^ High Frequency electromagnetic waves are the radio waves which are used in mobile communications.^6^, ^7^

The cochlea is the hearing unit where electrical hearing pathways, which consist of inner and outer vibratile ciliated cells, begin. The cochlear cells can be easily affected by a number of factors such as drugs, voice, systemic diseases, and trauma. The cells most susceptible to degeneration are outer vibratile ciliated cells. Auditory functions can be assessed directly by Otoacoustic Emission (OAE). Responses going backward from the cochlea and obtained at the outer ear canal show normal function.^8^

We are frequently exposed to magnetic field forces in many areas of daily life and they are also used for diagnosis and treatment purposes. The format of waves and the time of exposure to the waves are the same as the ones used for therapeutic purposes. The waves for diagnostic purposes are 50–200 MHz; that is very hıgh frequency. Whether it is effective or not on the cochlear cells is an issue to be researched.

In our study, we examined the effect on the cochlea of very low dose (40 Hz) magnetic field, which is used as a therapeutic dose in the literatüre. We applied in vivo pulsed low dose magnetic field to laboratory rats and then their cochlear function were examined by Distortion Product Otoacoustic Emission (DPOAE) test, and immunohistochemically.

## Materials and methods

### Animal care and experimental procedure

Twelve male Wistar albino rats weighing 250–300 g were used in our study. Prior to the experiments, ethics approval was received from the Local Ethics Committee for Animal Experiments (2017/611). Initially, the rats were left in the laboratory with a room temperature of 24–25 °C, humidity of 40%–50%, and 12 h night/day, in accordance with the circadian rhythm, for one week for the adaptation period. After one week of adaptation periods, the rats were separated into two groups, the study group (Group I, *n* = 6), which is exposed to the magnetic field, and the control group (Group II, *n* = 6). The rats at the study group were exposed to Pulsed Magnetic Field (PMF) of the same intensity and frequency (1.5 mT, 40 Hz) for 1 h at the same time of the day for 30 days. During this 1 month period the rats were ad libitum fed with food and water.

PMF was applied to rats by using a system with Helmholtz coils 60 cm in diameter, placed 30 cm apart. These coils in a Faraday cage were connected to a signal generator (ILFA Electronic, Adana, Turkey) and produced a magnetic field peak amplitude of 1.5 mT (1.49–1.51 mT). The peak value of the magnetic field was measured by a gauss meter with a Hall-effect probe (FW Bell model 6010, Sypris, Orlando, FL, USA). The time varying magnetic field consisted of a quasi-triangular waveform, with a rise time of 0.5 ms and a fall time of 9.5 ms. The induced electric field was a unipolar rectangular waveform having peak electric fields of 0.6 V/m (0.59–0.61 V/m) between the coils. PMF application period has been determined by examining and optimizing the studies in the literature in which PMF effect has been experienced.^9^, ^10^

### DPOAE test measurements

The animals were exposed to DPOAE twice, before the study began, and at the end of the 4 week experiment. The DP gram measurement was taken between 500 Hz and 8.000 Hz (used instrument: Neuro-Audio/OAE (version 2010) OAE probes: OAE probe (Neurosoft), ER-10D probe). The functional evaluation at that frequuencies 5–8 kHz, correspond to bazal turns. In the DPOAE, the ratio between the frequencies f2 and f1 (f2/f1) was set to 1.22, and the stimulus intensities were set to L1 = 55 and L2 = 55. DP grams were recorded by measuring for each octave. Six frequency points were sampled. DPOAE Signal-to-Noise Ratio (SNR) values were recorded at frequencies of 988 Hz, 1481 Hz, 2222 Hz, 2963 Hz, 5714 Hz and 8000 Hz. The SNR averages of the DPOAE responses of Group II which was not exposed to the magnetic field and Group I which was exposed to magnetic field was analyzed by using Kruskal–Wallis Test. Thus the groups were compared with each other.

### Histopathological examination

After examining the rat's hearing by DPOAE (Distortion Product Otoacoustic Emission) at the end of the four-week period, all the rats were sacrificed instantaneously at the same time by decapitation method after giving anesthesia with the combination of intraperitoneal ketamine hydrochloride 40 mg/kg (Ketalar-PFIZER) and xylazine hydrochloride 5 mg/kg (XylazinBio-BIOVETA). After the decapitation, the ears were removed and the formula was injected into the inner ear. Then the histological preliminary preparation was started by taking the cochleas after their transfer with 4% paraformaldehyde. The tissues were removed in formalin for 24 h. They were then placed in a 0.1 mol/L Ethylenediamine Tetra-Acetic Acid (EDTA) (Sigma–Aldrich, USA) solution for 3 weeks for the decalcification of osseous tissues. This was followed by an overnight washing under a water flow. After they were dehydrated through a graded ethanol series, they were cleared in xylene and processed for embedding in paraffin wax, according to routine protocols. The basal turn of the cochlea was evaluated. The figures presented correspond to basal turn.

### Immunohistochemical method

Paraffin blocks of the tissue were cut into 5 μm sections. For immunohistochemistry, sections were treated with 2% trypsin in Tris buffer (Sigma–Aldrich, St Louis, Missouri, USA) at 37 °C for 15 min. After treatment with 0.3% hydrogen peroxide in methanol for 15 min, sections were incubated in a blocking solution (Invitrogen, Carlsbad, California, USA) for 10 min. They were then incubated in a humid chamber for 1 h at 4 °C with primary antibody agonists Caspase-3 (rabbit polyclonal antibody; Lab vision, USA), Caspase-9 (rabbit polyclonal antibody, Lab vision, USA), all diluted to 1:100. Sections were then incubated with biotinylated secondary antibody and then with streptavidin conjugated to horseradish peroxidase for 30 min each (Invitrogen), following the instructions on the kit. Finally, they were incubated with 3-Amino-9-Ethylcarbazole (AEC) (Spring, California, USA), prepared according to the manufacturer's instructions, for 3–5 min, after which the nuclei were counterstained with Mayer's hematoxylin. The slides were visualized, and images were obtained using a photolight microscope (CX31 Olympus, Germany) attached to a digital camera (C-5060 Olympus, Germany). The figures were obtained from the basal turn of cochlea. Immunohistochemical analysis was performed on kidney cross-sections for all animals using image-analyzing software (Leica Q Win V3 Plus Image). Two independent observers blind to the treatment regimen performed separate immunolabeling score evaluations. Labeling intensity was graded semi-quantitatively, and the HSCORE was calculated using the equation HSCORE = Σ*Pi* (*i* + 1), where *i* is the intensity of labeling with a value of 1, 2 or 3 (weak, moderate or strong, respectively) and *Pi* is the percentage of labeled cells for each intensity, ranging from 0% to 100%.

### *TUNEL method* (terminal deoxynucleotidyl transferase dUTP nick end labeling)

The deparaffinized and rehydrated sections, prepared as described above, were stained using a commercial kit (Apoptag, S7101, Chemicon, CA, USA) in accordance with the manufacturer's instructions. The slides stained with a TUNEL technique were evaluated using a CX41 bright-field microscope (Olympus, Tokyo, Japan). Two observers blinded to experimental information evaluated the TUNEL scores independently. The number of all positive immunoreactive cells for all subjects was counted from apical to basal turn and analyzed. The average number of apoptotic cells was determined by counting TUNEL-positive cells which were counted in randomly chosen fields per case. In each case, a total of one hundred cells, either TUNEL-positive or negative, was calculated, and the percentage of TUNEL-positive cells was given. Cells in the areas of necrosis and poor morphology or at the borders of the sections were not included.

### Statistical analysis

Kruskal–Wallis test was used to compare the DPOAE responses and SNR averages of the groups. The Mann–Whitney *U* test was used with Caspase-3, Caspase-9 and TUNEL methods to compare the apoptosis status of the groups. The value of *p* less than 0.05 (*p* < 0.05) was considered to be significant.

## Results

The SNR (Signal-to-Noise Ratios) values at six frequency points were recorded and analyzed by each other at the DPOAE assessment using Kruskal–Wallis test. A statistically significant difference was determined (*p* < 0.05) at the 5714 Hz and 8000 Hz stimuli hearing thresholds and the SNR values were decreased in Group I (Table 1).

**Table 1:** Comparison of the mean and standard deviation (SD) of SNR averages of the DPOAE responses of the groups with Kruskal–Wallis test.

Groups	988 Hz	1481 Hz	2222 Hz	2963 Hz	5714 Hz	8000 Hz
Group I PMF (+)	3.72 ± 9.58	11.31 ± 11.48	7.28 ± 10.41	12.05 ± 5.66	21.58 ± 8.87	23.70 ± 6.29
Group II PMF (−)	3.96 ± 10.96	11.68 ± 10.32	7.35 ± 9.77	12.95 ± 5.86	27.38 ± 6.83	27.80 ± 10.71
*p*	0.190	0.213	0.132	0.97	<0.05	<0.05

The apoptotic cells were marked and counted using the TUNEL, Caspase 3 and Caspase-9 staining (Table 2).

**Table 2:** The cell counts marked using the TUNEL, caspase-3 and caspase-9 staining.

	Caspase 3						Caspase 9						TUNEL					
	1	2	3	4	5	6	1	2	3	4	5	6	1	2	3	4	5	6
Group 1 PMA (+)	161	178	166	164	177	179	193	195	169	194	180	189	12	8	10	13	20	7
Group 2 PMA (−)	131	112	123	135	117	128	145	140	143	137	142	153	2	1	2	3	1	4

**Figure 1:**
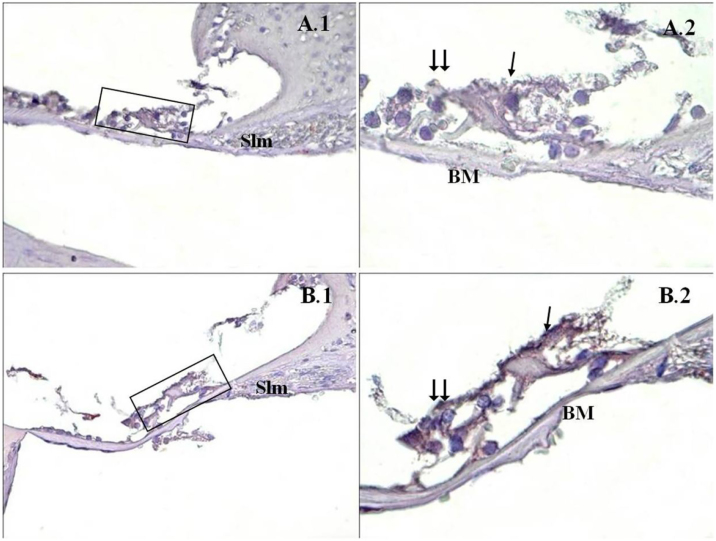
Caspase-3 immunostaining of cochlea corti organ. →: inner ciliated cells, → →: outer ciliated cells, Slm: spiral limbus, Slg: spiral ligament, BM: bazillar membrane, PMA (−) (A), PMA (+) (B), corti organ 40× (1), corti organı 100× (2). Mayer's hematoxylin background staining.

**Figure 2:**
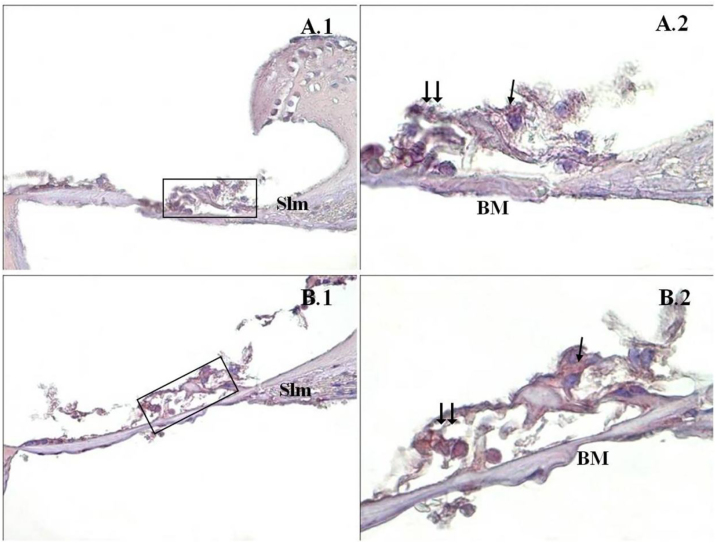
Caspase-9 immunostaining of cochlea corti organ. →: inner ciliated cells, → →: outer ciliated cells, Slm: spiral limbus, Slg: spiral ligament, BM: bazillar membrane, PMF(−) (A), PMF (+) (B), corti organ 40× (1), corti organ 100× (2). Mayer's hematoxylin background staining.

**Figure 3:**
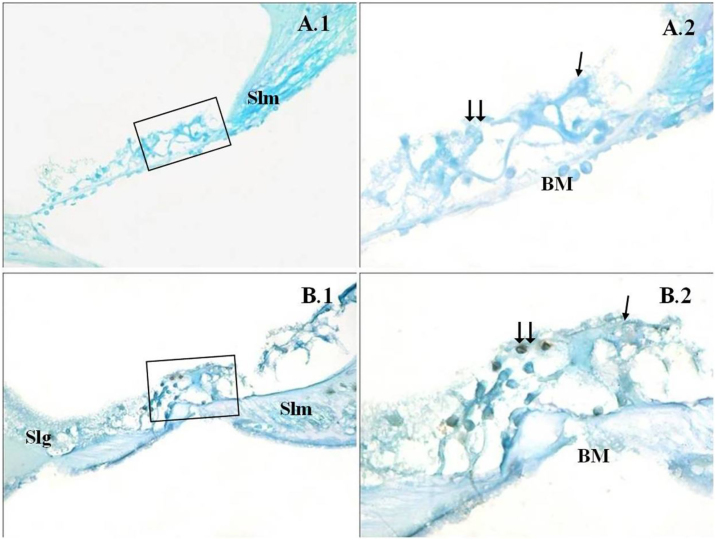
Cochlea corti organ, TUNEL method. →: inner ciliated cells, → →: outer ciliated cells, Slm: spiral limbus, Slg: spiral ligament, BM: bazillar membrane, PMF (−) (A), PMF (+) (B), corti organ 40× (1), corti organ 100× (2). Methyl green background staining.

A significant difference was found between the groups (*p* < 0.001) when the apoptosis results were compared by using Caspase-3, Caspase-9 and TUNEL methods (Table 3). While poor expression was observed in the outer ciliated cells of the corti organ of PMF (−) group during Caspase-3 immunostaining (Fig. 1A), moderate reaction was observed in the PMF (+) group (Fig. 1B) (*p* < 0.001). While reactions ranging from poor to moderate were observed in the outer ciliated cells of the corti organ of PMF (−) group during Caspase-9 immunostaining (Fig. 2A), immunostaining increase ranging from moderate to strong, especially at the outer ciliated cells of the corti organ, was observed in the PMF (+) group (Fig. 2B) (*p* < 0.001). While there were almost no Tunel-positive cells in the corti organ of the PMF (−) group which was stained by the TUNEL method (Fig. 3A), the outer ciliated cells and support cells of the corti organ in the PMF (+) group were determined to show Tunel positivity (Fig. 3B) (*p* < 0.001).

**Table 3:** Comparison of the mean of apoptotic cells of the groups, by using Mann–Whitney *U* Test.

	Group I – PMF (+) (*n* = 6)	Group II – PMF (−) (*n* = 6)	*p*-value
Caspase-3	170.83	124.33	<0.001
Caspase-9	186.66	143.33	<0.001
TUNEL	11.66	2.16	<0.001

## Discussion

In this study, we aimed to examine the changes in the otoacoustic emission, along with histopathological and immunohistochemical degenerations in the cochlea at the rats exposed to very low dose pulsed magnetic fields. We have seen statistically significant degeneration in cochlear cells both in the high-frequency values of DPOAE and histopathologically as a result of magnetic field exposure.

Biological systems are usually exposed to magnetic field forces at different intensities and frequencies, either directly or indirectly, in daily life. Many studies show that these forces have effect on the proliferation of the cell, synthesis and secretion of growth factors, programmed cell death, etc. These studies also mention their carcinogenic effect.^11^, ^12^ Likewise, the relationship between the magnetic field and neurogenic tumors, such as acoustic neurinomas and gliomas, has also been demonstrated.^13^ There are not many studies on its effects on the cochlea and cochlear cells. Whether magnetic field exposure has a negative effect on hearing is still controversial in the literature. Although some studies show that they do not cause any difference in hearing, some other studies show that they have negative effects.^14^, ^15^, ^16^, ^17^, ^18^

Continuous or intermittent magnetic field waves were used in the previous studies which were conducted to examine the negative effects of magnetic field. Studies about investigating the differences between continuous and pulsed EMF are insufficient. Increase in DNA damage and lipid peroxidation were determined at the studies applied with the magnetic field with continuous low-dose (50 Hz) frequency.^19^, ^20^ In another study made on rats by using pulsed magnetic field of 40 Hz, increase in IL-6, which is an inflammation marker, was determined in heart and brain cells.^21^

Despite these negative results, the therapeutic use of the low dose magnetic field has been found in recent studies. In the literature, 40 Hz magnetic field was used for wound healing, bone marrow edema and diabetic neuropathy for treatment purposes, and positive results have been reported.^2^, ^3^, ^4^ In another study, the rats were exposed to pulsed magnetic field of 50 Hz for 3 times a day for 4 h and it was found to reduce necrosis in urethral cells.^22^ For almost 50 years various forms of EMFs have been used to promote bone formation after fractures as well as for the treatment of osteoporosis.^5^ In an in vitro study, they found that increased osteoblastic activities were seen in very low dose pulsed EMF therapy in human osteoblast cells.^23^ Neuropathic pain remains difficult to treat. Due to the lack of effective therapeutic agents, it is necessary to search for potential alternative therapies for this kind of diseases. Recent studies have shown that magnetic application may have positive impact on the regeneration of injured peripheral nerves.^24^

The effect of pulsed very low dose EMF effects on cochlear cells is obscure. Previous studies examining cochlear damage were made at the magnetic field frequency which corresponds to the GSM frequency (300 MHz–300 GHz) and apoptosis in cochlear cells and degeneration of the cochlear nucleus were detected.^15^, ^16^, ^17^, ^18^, ^19^, ^20^, ^21^, ^22^, ^23^, ^24^, ^25^ When the use of low-frequency magnetic field for therapeutic purposes has become discussed in recent years, whether the low dose magnetic field causes damage to cochlear cells has become a research subject. In our study, we examined the effect of very low dose (40 Hz) magnetic field on the cochlea, which is used as a therapeutic dose in the literatüre. The mechanism of cochlear cell dejeneration in response to different ototoxic stimuli like magnetic field share a final common pathway means apoptosis. This programmed cell death also can be seen in normal cells and ends with phagostosis and no inflamation occurs. There may be less staining since there is a continuedapoptosis normally in the control group. However, in this study a significant difference was found between the groups (*p* < 0.001) when the apoptosis results were compared by using Caspase-3, Caspase-9.

We used DPOAE to measure the hearing functions in multiple frequencies at the rats. This measurement is a noninvasive, painless, practical, and objective measurement that does not require active participation.^26^, ^27^ Outer vibratile ciliated cells are important indicators of cochlear damage. Outer vibratile ciliated cell damage can be determined by using DPOAE. The rapid electrical potential change caused by magnetic field might disrupt homeostatic balances in cells with electrical signaling mechanisms by affecting signal transmission mechanisms. Also, studies which show that the application of magnetic field affects the activation of voltage-gated Ca^2+^ channels on the plasma membrane and Ca^2+^ which increases uncontrollably in the cells causes caspase activation through cytochrome c secreted from the mitochondria and it causes apoptosis.^28^ We used Caspase 3–9 and TUNEL methods to examine the cochlear apoptosis in our study. According to our study results, the values we detected with DPOAE in the group exposed to the magnetic field are correlated with our histopathological examination results. In summary increase of Ca^2+^ in the cell, increase in DNA damage and lipid peroxidation and increase of IL-6 all causes apoptosis and effect each other. Mechanisms of lesions of the EMF in cohlear hairy cells which lead to apoptosis mıght include all these situations and further studies and research into such biochemical reactions are indicated.

The major limitation of this study is that the auditory and histochemical results generated by EMF were examined only at the cochlear level. The cochlea is an important part of the auditory pathway and is very sensitive to external risk factors. Auditory brainstem response would have been a much better measure of auditory function with the aid of retrocochlear histopathology to evaluate the whole auditory pathway. In future studies, retrocochlear effects should be examined in addition to the cochlea.

## Conclusion

According to the results of our study, the very low dose magnetic field, which is considered to be used for various therapeutic purposes recently, can cause both auditory function defects and histopathologic damage in cochlear cells. Histological studies in human cochleas are difficult to obtain. Our study results were in rats and studies in humans, using OAE or ABR are still lacking. We think that more studies with various frequency magnetic field doses will contribute to the literature.

## Conflicts of interest

The authors declare no conflicts of interest.
